# Surgical transcatheter valve implantation: The more pathways, the better

**DOI:** 10.1111/jocs.16303

**Published:** 2022-02-09

**Authors:** Vito D. Bruno, Gianni D. Angelini

**Affiliations:** ^1^ Bristol Heart Institute Bristol University Bristol UK

**Keywords:** subclavian, TAVI, transapical

## Abstract

Transcatheter aortic valve implantation (TAVI) is rapidly spreading across the world with the endorsement of the cardiological community and the supporting results of randomized controlled trials. However, TAVI‐related complications like aortic dissection, aortic valvular rupture, or left ventricle perforation are still potentially catastrophic.

Transcatheter aortic valve implantation (TAVI) is rapidly spreading across the world with the endorsement of the cardiological community^1^ and the supporting results of randomized controlled trials.^2^, ^3^ However, TAVI‐related complications like aortic dissection, aortic valvular rupture, or left ventricle perforation are still potentially catastrophic.^4^

At the same time, the presence of small or tortuous femoral and iliac arteries might represent a limiting factor for percutaneous approaches^5^ with the most common obstacle being narrowed luminal diameter as the artery must be at least 1.25% of the sheath's outer diameter for safe insertion.^6^ Therefore, with the growing number of TAVI implantations, the availability of a surgeon during these procedures becomes increasingly relevant, especially in high‐risk or complex cases. Surgeons can provide different types of surgical approaches for TAVI with transapical (TA) and subclavian (SC) being the most frequently used.^7^ In this issue of the *Journal of Cardiac Surgery*, D'Auria et al.^8^ compared these two approaches in a large nationwide analysis involving more than 1500 patients in 36 UK‐based centers, demonstrating the safety and reproducibility of both techniques as valid alternatives to the classic femoral approach. Their series includes high‐risk patients with an elevated Logistic Euroscore and a median age of 80 years. Despite this high‐risk cohort, the authors reported remarkable clinical results with both surgical approaches. The complications rates were similar between groups, although in hospital mortality was higher in the TA group (6.9% vs. 1%; *p* = .04). This might reflect a slightly riskier population in this group or an older era of implantation, being the TA‐TAVI conducted earlier than the SC route. Another difference was the need for permanent pacemaker (PPM) higher in the SC group (28% vs. 11%; *p* = .02), although it is difficult to associate this finding with the surgical approach used. Most importantly, the operative characteristics and vascular complications were comparable between the groups and although the surgical operative time was longer with the SC approach, this group had a significantly shorter length of stay in hospital. Interestingly the 8‐years follow survival was similar in the two groups which is reassuring on the efficacy of these treatments regardless of the surgical approach in a high‐risk population. Another important finding of the paper was related to the progressive improvement of the survival rates over the years, as we can see in the proportional hazard model shown in table 3. The more recent year of implantation was a protective factor in terms of long‐term survival, reflecting the progressive clinical and technological advancement of these procedures and the improved experience of those performing them. There are limitations in this study mainly represented by its retrospective and nonrandomized nature, although this was partially mitigated by a propensity score‐matched analysis. Another limitation was the absence of different surgical approaches like the transaortic that can be performed via a mini‐sternotomy or thoracotomy and is a valid alternative in case of difficult peripheral accesses or reduced lung function.^9^ This is not the first study from the UK TAVI registry and in 2015, a 2 years follow‐up was published.^10^ Interestingly, in that report, the SC access had similar survival than transfemoral, while TA and direct aortic approaches were worst. In another Italian study^5^ involving 874 consecutive patients from three different centers, the survival rates between TA and SC were also similar at 1 and 2 years follow‐up, although the in‐hospital short‐term mortality was again slightly higher in the TA group (8.5% vs. 1.7%; *p* = .06).^5^ A recent meta‐analysis^11^ confirmed these results, showing a lower early all‐cause mortality in the SC group, but with a higher incidence of pacemaker implantation (odds ratio: 4.22; *p* = .0001) compared to TA.^11^ The same study showed a worst midterm survival in SC when compared to the transfemoral approach. In another retrospective analysis of transthoracic (TA and transaortic) versus transvascular (SC and carotid) approaches, the latter groups had a better short‐term outcome and a shorter hospital length of stay.^12^ Moreover, another meta‐analysis has shown that SC access is a safe and feasible alternative access route for TAVI with lower risks of major vascular complications.^13^ It is important to note that despite its satisfying short‐ and long‐term results and the potential financial benefits, the SC access is still rarely used and represents only a minor part of the surgical TAVI procedures^14^ and in D'Auria's study only 290 SC (vs. 1 216 TA) TAVIs were reported. In our opinion, this surgical site represents an effective alternative in patients with contraindication to both transfemoral and TA techniques and it can also be approached percutaneously in selected cases.^15^ D'Auria and colleagues should be congratulated for providing evidence that surgical TAVIs can be safely done even in high‐risk patients. Cardiac surgeons have a variety of surgical approaches in their portfolio which can expand the use of TAVI in patients with technical contraindications to the standard femoral approach. Moreover, surgeons can quickly address major cardiovascular complications that might arise during or after the TAVI procedure. In this context, the benefit of an effective and well‐integrated heart team is of a particular relevance. A close collaboration between cardiologists and cardiac surgeons is of paramount importance to treat the increasingly complex variety of heart valve disease.

## CONFLICT OF INTERESTS

The authors declare that there are no conflict of interests.
